# Comparison between clinical and audiological results of tympanoplasty with modified sandwich technique and underlay technique^☆^

**DOI:** 10.1016/j.bjorl.2017.03.009

**Published:** 2017-04-21

**Authors:** Sanjana Vijay Nemade, Kiran Jaywant Shinde, Chetana Shivadas Naik, Haris Qadri

**Affiliations:** Smt. Kashibai Navale Medical College and General Hospital, Pune Maharashtra, India

**Keywords:** Tympanoplasty, Temporalis fascia graft, Areolar fascia graft, Sandwich technique, Post operative hearing gain, Timpanoplastia, Enxerto de fáscia temporal, Enxerto de fáscia aureolar, Técnica “sanduíche”, Ganho auditivo pós-operatório

## Abstract

**Introduction:**

Surgical repair of the tympanic membrane, termed a type one tympanoplasty is a tried and tested treatment modality. Overlay or underlay technique of tympanoplasty is common. Sandwich tympanoplasty is the combined overlay and underlay grafting of tympanic membrane.

**Objective:**

To describe and evaluate the modified sandwich graft (mediolateral graft) tympanoplasty using temporalis fascia and areolar fascia. To compare the clinical and audiological outcome of modified sandwich tympanoplasty with underlay tympanoplasty.

**Methods:**

A total of 88 patients of chronic otitis media were studied. 48 patients (Group A) underwent type one tympanoplasty with modified sandwich graft. Temporalis fascia was underlaid and the areolar fascia was overlaid. 48 patients (Group B) underwent type one tympanoplasty with underlay technique. We assessed the healing and hearing results.

**Results:**

Successful graft take up was accomplished in 47 patients (97.9%) in Group A and in 40 patients (83.3%) Group B. The average Air-Bone gap closure achieved in Group A was 24.4 ± 1.7 dB while in Group B; it was 22.5 ± 3.5 dB. Statistically significant difference was found in graft healing rate. Difference in hearing improvement was not statistically significant.

**Conclusion:**

Double layered graft with drum-malleus as a ‘meat’ of sandwich maintains a perfect balance between sufficient stability and adequate acoustic sensitivity.

## Resumo

**Introdução:**

O reparo cirúrgico da membrana timpânica, denominado timpanoplastia tipo 1, é uma modalidade de tratamento já bem estabelecida. As técnicas *overlay* ou *underlay* de timpanoplastia são comuns. A timpanoplastia pela técnica “sanduíche” é a técnica de enxerto de membrana timpânica *overlay* e *underlay* combinadas.

**Objetivo:**

Descrever e avaliar a timpanoplastia com a técnica “sanduíche” modificada (timpanoplastia mediolateral) utilizando fáscia temporal e fáscia aureolar. Comparar o desfecho clínico e audiológico da timpanoplastia com a técnica “sanduíche” modificada com o da timpanoplastia com a técnica *underlay*.

**Método:**

Foram estudados 88 pacientes com otite média crônica. 48 pacientes (Grupo A) foram submetidos à timpanoplastia tipo 1 com enxerto “sanduíche” modificado. A fáscia temporal foi utilizada na técnica *underlay* e a fáscia areolar na técnica *overlay*. 48 pacientes (Grupo B) foram submetidos à timpanoplastia tipo 1 com a técnica *underlay*. Foram avaliados os resultados da cicatrização e da audição.

**Resultados:**

O sucesso do enxerto ocorreu em 47 pacientes (97,9%) no Grupo A e em 40 pacientes (83,3%) do Grupo B. O fechamento médio do gap aéreo-ósseo no Grupo A foi de 24,4 ± 1,7 dB, enquanto no Grupo B foi de 22,5 ± 3,5 dB. Houve diferença estatisticamente significativa na taxa de cicatrização do enxerto. A diferença na melhora auditiva não foi estatisticamente significante.

**Conclusão:**

O enxerto de camada dupla e o tímpano-martelo posicionados como o “recheio” do sanduíche mantém um equilíbrio perfeito entre a estabilidade necessária e adequada sensibilidade acústica.

## Introduction

Chronic otitis media with perforation of the tympanic membrane is a common cause of hearing loss and ear discharge.^1^ There are two popular surgical techniques, the underlay and overlay methods for tympanoplasty. The underlay technique is quicker and easier to perform, and the creation of a tympano-meatal flap with elevation of the annulus allows inspection of the ossicular chain.^2^ However, there is a risk of medial displacement of the graft, especially in large and/or anterior perforations.^3^ The overlay technique avoids this pitfall, but there is a risk of keratin pearl formation within the tympanic membrane, and also a risk of blunting of the angle between the drum and the anterior meatal wall.^2^ A number of other techniques of tympanic membrane repair have been described. The term ‘sandwich technique’ was coined by Farrior in 1983 to describe a method in which sheets of areolar fascia were placed medial and lateral to the drum, with the fibrous layer as the ‘meat’ in the sandwich.^4^, ^5^ Raghavan et al. used the same term to describe a technique in which a pedicle skin flap is used to partially cover an overlay tympanic membrane graft of temporalis fascia.^6^ We have modified the sandwich graft by using two different graft materials, i.e. temporalis fascia and areolar fascia; and drum-malleus is sandwiched between the two. We compared the healing and hearing results of tympanoplasty with underlay technique and modified sandwich technique.

## Methods

We prospectively studied 96 patients during 2014–2016. Institutional Review Board approval was taken. The approval protocol number is Ref. SKNMC No/Ethics/App/2014/236 dated 23/07/2014. The Registration number is ECR/275/Inst/MH/2013.

### Objectives

1. To compare the clinical outcome in terms of healing of the graft in tympanoplasty by underlay technique and modified sandwich technique.
2. To compare the audiological outcome in terms of post operative hearing gain in tympanoplasty by underlay technique and modified sandwich technique.

### Inclusion criteria

Patients with tubotympanic type of chronic otitis media with large or subtotal perforation.

### Exclusion criteria

Patients with atticoantral type of chronic otitis media, revision ear surgery, patients requiring ossicular reconstruction, patients with mixed hearing loss.

Group A (*n* = 48) includes patients who underwent tympanoplasty by modified sandwich technique.

Group B (*n* = 48) includes patients who underwent tympanoplasty by underlay technique. Data collected of each patient included: age, gender, previous ear surgery, preoperative pure tone audiometry and clinical findings, surgical details, postoperative clinical findings and pure tone audiometry. In all cases the ear was dry and with normal middle ear mucosa for at least one month prior to surgery.

### Technique

**Figure 1:**
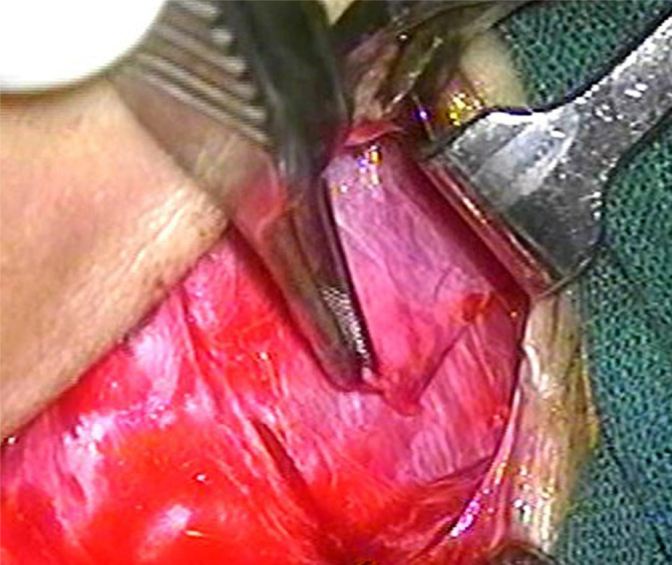
Areolar fascia harvesting.

**Figure 2:**
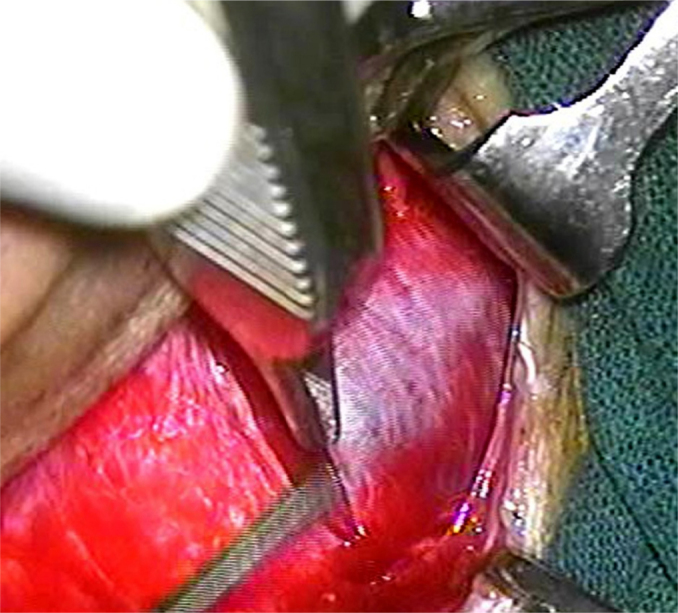
Temporalis fascia harvesting.

**Figure 3:**
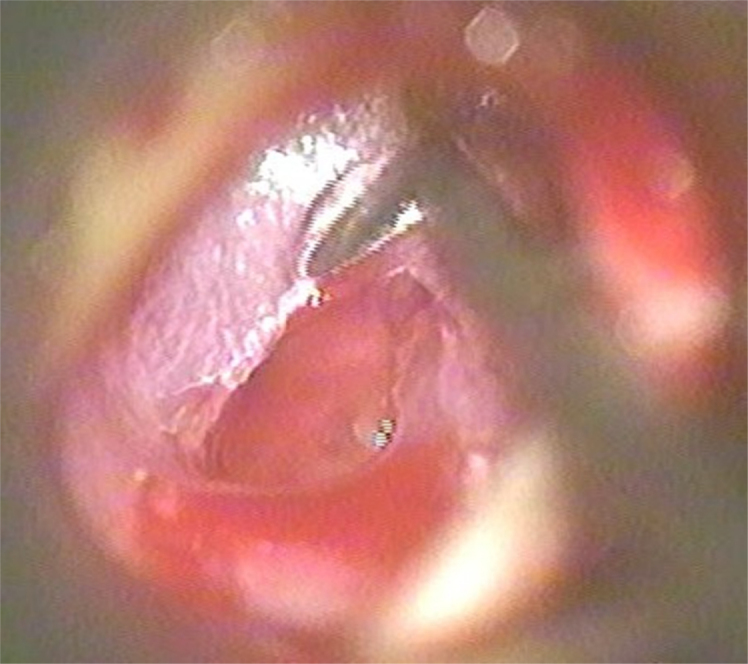
Removing the epithelial layer.

**Figure 4:**
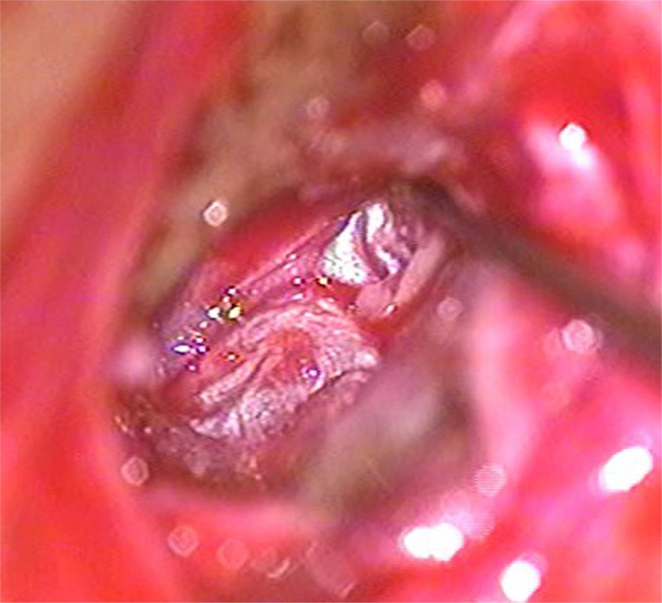
Temporalis fascia underlaid.

**Figure 5:**
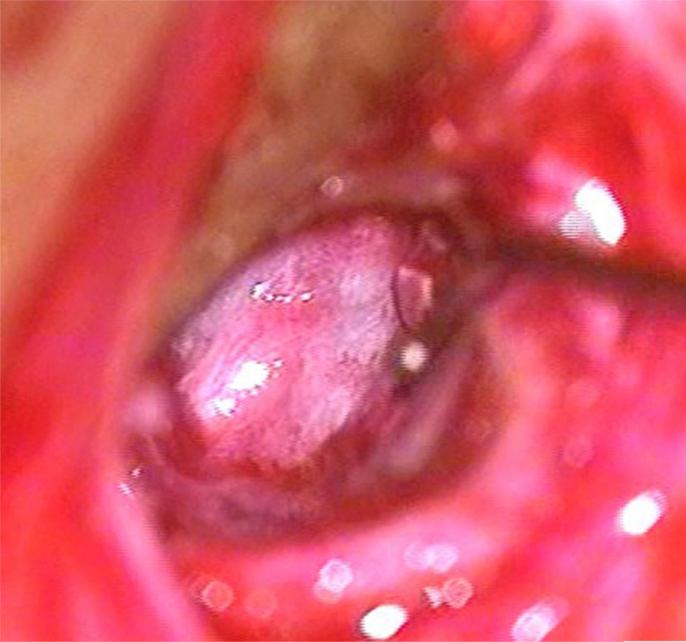
Areolar fascia overlaid.

A post-auricular approach was used under intravenous sedation supplemented with local infiltration of 2% Xylocain with 1:200,000 adrenaline. Areolar fascia (Fig. 1) and temporalis fascia (Fig. 2) was harvested. In underlay technique, only temporalis fascia was harvested. Posterior meatotomy was done. The edges of the perforation were scrupulously denuded to promote good capillary blood flow. Mucosal surface of tympanic membrane was freshened with Rosen's knife to create a raw undersurface. In modified sandwich technique, epithelial layer of anterior part of tympanic membrane was removed upto the annulus to create a raw surface laterally (Fig. 3). Epithelial layer of the posterior aspect of tympanic membrane was kept intact. The handle of malleus was also denuded off the epithelium. In underlay technique, we do not need to remove epithelium of the anterior part of tympanic membrane. Vascular stripe incision was taken and posterior tympanomeatal flap was elevated. The middle ear was exposed. Ossicular chain mobility was confirmed. In underlay technique, temporalis fascia was underlaid medial to handle of malleus and the tympanomeatal flap was reposited. In modified sandwich technique, temporalis fascia graft was underlaid medial to the handle of malleus (Fig. 4). Now the areolar fascia graft was overlaid lateral to handle of malleus and the fibrous layer of tympanic membrane (Fig. 5). The tympanomeatal flap was then reposited. Trapping the epithelium and skin beneath the graft was avoided. Thus the handle of malleus and the fibrous layer of tympanic membrane was sandwiched between the two grafts only in the anterior aspect, whereas in the posterior aspect, the graft was lateral to malleus and medial to tympanic membrane. As the chances of medialisation or lateralization of the graft are found to be more in the anterior aspect, this sandwich in the anterior aspect was expected to provide adequate stability to the graft. Gelfoam was placed over the graft for stabilization. The post-auricular incision was closed in two layers and mastoid dressing was applied.

A successful anatomical outcome was considered to comprise full, intact healing of the graft without perforation, retraction, lateralization or blunting post-operatively and with improvement of hearing. Graft healing was evaluated in all patients and postoperative complications were noted. Average preoperative Air-Bone gap, postoperative Air-Bone gap and the Air-Bone gap closure in dB at 500 Hz, 1000 Hz, 2000 Hz were noted.

### Post-operative care

Antibiotics were given for 5 days. Suture removal was done one week after surgery and the gelfoam was suctioned from the ear canal 3 weeks post-operatively. Antibiotic steroid-containing drops were used for a further 2 weeks to clear the residual gelfoam which can lead to granulation and fibrous tissue formation if not completely removed from the tympanic membrane. An audiogram was performed 3 months after surgery in patients with healed graft. The ear was examined at 6 months and, thereafter, every year.

## Results

The mean age was 34.3 ± 7.9 years with a range of 15–45 years. The male to female ratio was 1:0.76. In Group A (*n* = 48), we observed a successful graft take up in 47 patients (97.9%). One patient (2.1%) had postoperative infection and rejection of graft. In Group B (*n* = 48), 40 patients (83.3%) had well accepted graft. Five patients (10.5%) had rejection of graft due to medialisation and 3 patients (6.2%) had reperforation of graft due to post operative infection. Statistical comparative analysis was done for the graft healing rate of the two groups. Chi-square value with Yates correction was 4.414 with 1 degrees of freedom. Two tailed *p*-value was 0.0356. With Fisher's exact probability test, 2 tailed *p*-value was 0.0356. Statistically significant difference was found in the graft healing rate of the two groups (*p* = 0.0356) (Table 1).

**Table 1:** Analysis of graft healing.

Graft status on follow up	Group A (*n* = 48)	Group B (*n* = 48)
*1st (7th post-op day)*		
Intact	48 (100%)	47 (97.9%)
Rejected	0 (0%)	1 (2.1%)

*2nd (1 month post op)*		
Intact	47 (97.9%)	41 (85.4%)
Rejected	1 (2.1%)	7 (14.6%)

*3rd (2 months post op)*		
Intact	47 (97.9%)	40 (83.3%)
Rejected	1 (2.1%)	8 (16.4%)

*4th (3 months post op)*		
Intact	47 (97.9%)	40 (83.3%)
Rejected	1 (2.1%)	8 (16.4%)

Group A, modified sandwich tympanoplasty; Group B, underlay tympanoplasty.

Graft take up rate is Group A is 97.9%. Graft take up rate is Group B is 83.3%.

Chi-square value with Yates correction is 4.414 with 1 degrees of freedom. Two tailed *p*-value = 0.0356. With Fisher's exact probability test, 2-tailed *p*-value is 0.0356.

Statistically significant difference is observed.

In Group A, the preoperative average Air-Bone gap in speech frequencies (500 Hz, 1000 Hz, 2000 Hz) was 41.0 ± 3.9 dB and the post-operative average Air-Bone gap was 16.6 ± 2.6 dB. Average Air-Bone gap closure (dB) achieved was 24.4 ± 1.7 dB. In Group B, the preoperative average Air-Bone gap in speech frequencies (500 Hz, 1000 Hz, 2000 Hz) was 43.6 ± 4.4 dB and the post-operative average Air-Bone gap was 21.0 ± 4.6 dB. Average Air-Bone gap closure (dB) achieved was 22.5 ± 3.5 dB. Statistical analysis was done with Paired *t*-test. The *t* value was 0.9074 with standard error of difference 1.873. With Confidence Interval 95%, two tailed *p*-value was 0.460. The post operative hearing gain assessed after healed graft in the two groups was statistically not significant (Table 2).

**Table 2:** Analysis of hearing results.

	Preoperative average Air-Bone gap (dB)		Post-operative average Air-Bone gap (dB)		Average Air-Bone gap closure (dB)	
	Group A (*n* = 48)	Group B (*n* = 48)	Group A (*n* = 48)	Group B (*n* = 48)	Group A (*n* = 48)	Group B (*n* = 48)
500 Hz	36.5	38.5	13.6	16.7	22.9	21.8
1000 Hz	43.9	46.9	17.5	20.5	26.4	26.4
2000 Hz	42.7	45.4	18.7	25.9	24.0	19.5
Mean ± SD	41.0 ± 3.9	43.6 ± 4.4	16.6 ± 2.6	21.0 ± 4.6	24.4 ± 1.7	22.5 ± 3.5
SD	3.971	4.479	2.666	4.623	1.789	3.513
Variation of SD	15.77	20.07	7.112	21.37	3.20	12.34

SD, standard deviation.

Paired *t*-test: *t* value is 0.9074; df = 2 with standard error of difference 1.873.

With 95% confidence interval, two tailed *p*-value is 0.460.

Statistically no significant difference is observed in post operative hearing gain in two groups.

## Discussion

Successful tympanoplasty depends on the integrity and stability of tympanic membrane (TM) which in turn affects the final position of the reconstructed tympanic membrane. Though a variety of materials like skin, perichondrium, vein, dura and cartilage are available for closure of TM perforations, temporalis fascia is the most commonly used graft with its certain advantages as it is easily available in sufficient quantity and through same incision, its thickness is similar to TM with low basal metabolic rate.^7^ Final position of the graft depends upon the pressure changes in the middle ear and external ear. Normal tympanic membrane can withstand the pressure changes due to its integrity and stability. At the same time, the sound conduction is also optimal. An ideal graft should find a perfect balance between the stability as well as sound conduction.^7^ A single layered temporalis fascia graft has thickness similar to that of tympanic membrane thus giving optimal sound conduction. But the pressure changes in the middle ear can lead to medialisation or lateralization of the graft leading to failure of Tympanoplasty. Due to this reason, the average rate of successful tympanoplasty varies between 85% and 90%.^7^, ^8^

The sandwich graft tympanoplasty, described by Farrior,^4^ is double layer technique in which both a medial and lateral layer of areolar fascia are used. It has been shown to be highly effective in restoring the integrity of the tympanic membrane.^4^ We have modified this by using two different graft materials, i.e. temporalis fascia and the areolar fascia which is expected to provide a perfect balance between the stability and the acoustic sensitivity of the tympanic membrane. Temporalis fascia is underlaid and areolar fascia is overlaid. Thus the Fibrous layer of tympanic membrane and the handle of malleus are sandwiched between the two. Thickness of graft is always the concern for the audiological outcome of the surgery.^6^, ^7^ Areolar fascia being a thin connective tissue does not add to the thickness to an extent to hamper the sound conduction.

The sandwich tympanoplasty or the over-under tympanoplasty is a combination of the underlay and overlay techniques and has been developed with the aim of minimizing the disadvantages inherent in the other two techniques. This may explain why the sandwich graft is becoming popular.^1^, ^9^ There are a few studies on this relatively new technique in the literature. Stage and Bak-Pedersen^10^ who supported the over-under procedure when used for perforations anterior to the handle of the malleus, reported a success rate of 91% in 39 ears. A similar success rate (90%) was attained by Kartush et al.^9^ in a series of 120 patients who underwent over-under tympanoplasty. It was reported as 90% by Imran et al.^11^ Mills^1^ reported it as 97% with hearing improvement in 98% patients in his study of 123 patients. In all these techniques temporalis fascia has been used for double layer of sandwich. With modified Sandwich technique, we could achieve success rate of 97.9% in healing of graft, while that in underlay technique it was 83.3%, thus giving statistically significant difference (*p* = 0.0356) (Table 1).

The average Air-Bone gap closure which gives an indication of the degree of hearing improvement was 24.4 ± 1.7 dB in Group A and 22.5 ± 3.5 dB in Group B (Table 2). This implies that many patients had a useful improvement in the hearing and obtained a dry ear too. She et al.^12^ described hearing improvement of 9.7 dB in the over-under tympanoplasty (*n* = 30). In the study done by Yagit et al.^13^ it was 16.96 dB (*n* = 58). Another study by Ahmed et al.^14^ using mediolateral graft showed a hearing improvement of 12.65 dB.

As we experienced, the advantages of modified sandwich tympanolasty are:

1. Stability of the graft, like a button in a button hole.

2. Prevents medialisation or lateralization of graft.

3. Temporalis fascia and areolar fascia both can be harvested through the same incision.

4. Easy to perform because epithelial layer of only the anterior half of tympanic membrane remnant is elevated rather than the entire TM.

5. Though it is a double layer of graft, thickness is optimum for acoustic sensitivity.

## Conclusion

Single-layer graft techniques in tympanoplasty (especially in large and subtotal perforation) have persistent problems like fascia graft medialisation, lateralization and recurrent perforations. A double layered graft with temporalis fascia (underlay) and areolar fascia (overlay) using drum-malleus sandwich technique gives excellent results in postoperative healing of graft. Considerable audiological outcome is also achieved. This modified sandwich graft technique, and its results are presented to help the practicing otologic surgeon obtain a better understanding of this effective tympanoplasty.

## Conflicts of interest

The authors declare no conflicts of interest.
